# Study on the Lost Circulation Mechanism of Polymer-Based Drilling Fluid Systems in Deep Fractured Shale

**DOI:** 10.3390/polym17212929

**Published:** 2025-10-31

**Authors:** Yanbin Zang, Zengwei Chen, Yi Wang, Yan Zhang, Shengchi Xu, Junyu Xie, Wei Chen

**Affiliations:** 1 State Key Laboratory of Shale Oil and Gas Enrichment Mechanisms and Effective Development, Beijing 100083, China; 2 School of Petroleum Engineering, Yangtze University, Wuhan 430113, China; 3 Research Institute of Geology, CNPC Xibu Drilling Engineering Company Ltd., Karamay 834000, China

**Keywords:** shale, drilling fluid loss, fracture-induced lost circulation, loss rate, polymer-based drilling fluid

## Abstract

To elucidate the lost circulation mechanism in naturally fractured shale, this study employs fluid seepage theory and fracture deformation theory, assumes the polymer-based drilling fluid system behaves as a Herschel–Bulkley (H–B) fluid, and develops a calculation model for lost circulation pressure that comprehensively incorporates fracture geometry, fracture stress state, drilling fluid properties, and the pressure differential between the wellbore and the formation. Research shows that the lost circulation rate of drilling fluid increases with greater initial fracture width, fracture deformation index, fluid consistency coefficient, yield stress, and pressure differential between the wellbore and the formation, while it decreases with increasing fracture radial extension length, fracture roughness, drilling fluid density, and normal stress on the fracture surface. The initial fracture width, fracture radial extension length, and fluid consistency coefficient have a significant influence on the lost circulation rate of drilling fluid. In contrast, the effects of the fracture deformation index and dynamic yield stress are relatively minor, indicating that they are not the primary controlling factors of fracture-induced lost circulation.

## 1. Introduction

In recent years, with the gradual depletion of conventional oil and gas resources worldwide, unconventional resources such as tight oil and gas and shale oil and gas reservoirs have become the main focus of global oil and gas exploration and development [1,2,3,4]. During the development of shale oil and gas reservoirs, drilling fluid loss is inevitable regardless of whether water-based or oil-based drilling fluids are used [5,6,7]. Under the action of the pressure differential between the wellbore and the formation, drilling fluid flows from the wellbore into the formation, and when sufficient space exists to accommodate the fluid entering the formation, significant lost circulation occurs [8]. Drilling fluid loss not only increases drilling fluid consumption and raises drilling costs, but may also invade formation pores or fractures, causing formation instability. Moreover, the drilling fluid retained in the reservoir can pollute the subsurface environment [9,10,11,12].

To prevent and control drilling fluid loss, numerous scholars have conducted extensive research. Li et al. [13] conducted an in-depth analysis of the lost circulation mechanism of oil-based drilling fluids in shale oil drilling and found that shale oil reservoirs are characterized by well-developed natural fractures and high rock brittleness, making them prone to induced fracture-related fluid loss. Wang et al. [14], in order to clarify the lost circulation mechanism, established lost circulation pressure models for pressure-induced fracture loss, fracture-extension loss, and large-fracture/cavernous loss. Li et al. [15], based on single-well logging data and seismic interpretation data, investigated the response relationships among formation fractures, in situ stress, formation pressure, and drilling fluid loss. Their results indicate that pressure-differential loss through open fractures is closely related to fracture development and formation pressure, whereas loss due to the propagation of closed fractures is primarily related to in situ stress. Majidi et al. [16], based on the Herschel–Bulkley rheological model, established a natural fracture lost circulation pressure model and pointed out that lost circulation can be controlled by adjusting the drilling fluid density. Zhai et al. [17], combining the steady-state diffusion equation of bottomhole fluid and considering factors such as loss velocity coefficient, fracture width, fluid viscosity, and wellbore radius, developed a dynamic model of lost circulation pressure for steady-state fluid diffusion in fractures, demonstrating that controlling the lost circulation pressure to reduce fracture width can effectively mitigate fluid loss. Li et al. [18] established a two-dimensional rough fracture model based on fractal theory and used the Herschel–Bulkley model to describe the non-Newtonian rheological behavior of drilling fluids. It can be seen that drilling fluid leakage is the result of multiple factors. Formation geological characteristics, rock mechanical properties, formation pressure distribution and drilling fluid performance will lead to drilling fluid leakage.

To clarify the lost circulation mechanism in deep fractured shale and promote the sustainable development of oil and gas exploration, this paper, based on the traditional fracture loss model, comprehensively considers the effects of the polymer fluid yield stress, fracture roughness and conductivity variation, as well as the filtrate seepage process on the drilling fluid loss rate. It investigates the dynamic characteristics of drilling fluid flow within natural fractures, analyzes the influence of fracture parameters, fracture stress state, drilling fluid properties, and the pressure differential between the wellbore and the formation on fracture-induced lost circulation, and classifies the types of fluid loss under different parameter conditions. This work provides an effective approach for lost circulation prevention and plugging in deep fractured shale formations.

## 2. Analysis of the Mechanical Properties of Maokou Formation Shale

The Maokou Formation shale is distributed in the central and eastern parts of the Sichuan Basin and is one of the important shale gas reservoirs in China. The shale samples used in this study were collected from outcrops of the Maokou Formation. XRD experiments were conducted to determine the main mineral composition of the shale, revealing that the total clay mineral content ranges from 32.84% to 55.8%, while the quartz content ranges from 30.8% to 43.87%. The total brittle mineral content ranges from 30.8% to 50.12%, while the feldspar content is relatively low, ranging from 0% to 5.7%. During drilling, the lost drilling fluid primarily undergoes hydration reactions with illite and montmorillonite in the clay minerals, thereby weakening the mechanical properties of the rock [19,20,21]. Among them, montmorillonite mainly undergoes swelling reactions upon contact with water, exhibiting strong hydration-induced swelling forces. Illite, on the other hand, contains water molecules within its structure that interact with the water in the drilling fluid and undergo hydration reactions. Compared with montmorillonite, the hydration swelling capacity of illite is much weaker and can be considered almost negligible. To clarify the type of hydration reactions in the Maokou Formation shale, clay mineral content analysis was conducted on ten groups of shale samples, as shown in Table 1. The results indicate that the clay mineral content in the Maokou Formation shale is predominantly composed of non-swelling illite, suggesting that the effect of hydration swelling on the mechanical properties of the rock can be considered negligible.

To investigate the changes in the mechanical properties of the Maokou Formation shale after contact with water-based drilling fluids, uniaxial compression tests were conducted on shale samples subjected to different water immersion durations. Due to the anisotropy of the rock, in order to minimize its influence on the experimental results, all shale samples in this study were taken from the same shale outcrop. First, the samples were processed into cylindrical specimens with both height and diameter of 25 mm, followed by porosity and permeability tests. The porosity and permeability results are shown in Table 2. It can be seen that the selected Maokou Formation shale samples have an average porosity of 1.32% and an average permeability of 0.018 × 10^−3^ μm^2^. The relative deviations of porosity and permeability among the samples are within an acceptable range, indicating that the selected shale samples can be considered homogeneous.

**Figure 1:**
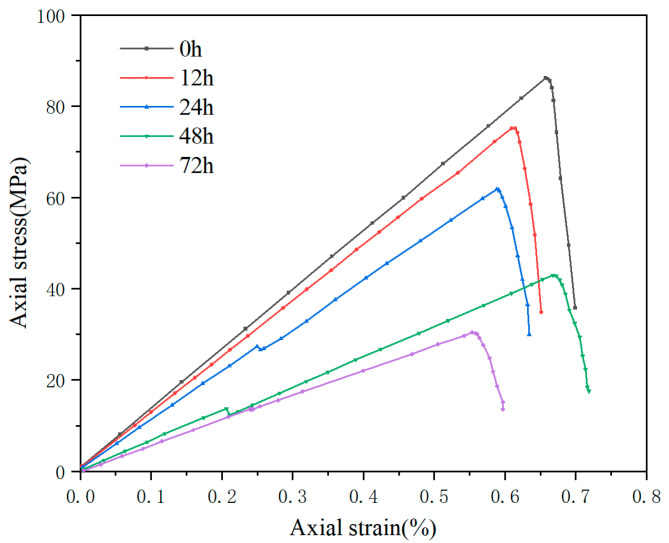
Stress–strain curves of shale samples in each group.

In the uniaxial compression tests, the samples were processed into cylindrical specimens with a height of 50 mm and a diameter of 25 mm. They were soaked for 0 h, 12 h, 24 h, 48 h, 72 h, and 96 h, respectively. The stress–strain curves for each group of shale samples are shown in Figure 1. It can be observed that, prior to contact with the drilling fluid, the Maokou Formation shale exhibits a distinct elastic–brittle failure characteristic. During drilling, large areas of induced fractures are easily formed in the formation surrounding the wellbore at the moment the drill bit breaks the rock [22,23,24]. Under the continuous seepage of drilling fluid, these fractures are highly likely to induce significant block falls, which can lead to wellbore instability and other related accidents. After contact with the drilling fluid, the uniaxial compressive strength and elastic modulus of the Maokou Formation shale decrease with increasing soaking time in the water-based drilling fluid. The drilling fluid penetrates into the rock and undergoes hydration reactions with the clay minerals in the shale, thereby weakening the rock’s compressive strength and elastic modulus and exerting a negative impact on formation stability [25,26,27,28,29]. At the same time, it can also trigger a series of accidents such as wellbore instability and blowouts, which not only prolong the drilling period but may also pose a serious threat to the safety of drilling personnel [30]. Therefore, clarifying the lost circulation behavior of drilling fluids in natural fractures of the target formation is of great significance for the subsequent on-site lost circulation prevention and plugging technologies, as well as for achieving efficient and safe drilling operations.

## 3. Calculation Model of Lost Circulation Pressure in Natural Fractures

### 3.1. Model Establishment

Based on the traditional fracture loss model, the proposed model considers the effects of the polymer fluid yield stress, the variation in fracture conductivity with pressure, and the filtrate seepage process, to characterize the flow and loss behavior of drilling fluids in deep fractured shale formations, thereby providing a more comprehensive representation of the actual seepage characteristics of drilling fluids in rough fractures. The roughness of fracture surfaces is one of the key factors causing deviations between the actual and theoretical seepage of drilling fluid in fractures, particularly when the fracture aperture is small, making surface roughness especially significant [31,32,33,34]. Therefore, a proper evaluation and characterization of fracture surface roughness play a crucial role in the study of fluid flow in rough fracture structures.

In this study, fracture roughness is characterized using hydraulic aperture, mechanical aperture, and fracture tortuosity. The relationship between fracture hydraulic aperture, mechanical aperture, and fracture tortuosity is expressed as follows:

(1) $$w_{m}=w_{h}\delta^{\frac{1}{3}}$$

where $w_{m}$ is the mechanical aperture of the fracture (m), $w_{h}$ is the hydraulic aperture of the fracture (m), and δ is the fracture tortuosity.

The fracture tortuosity ranges from 1 to 2. When the tortuosity equals 1, the fracture surface is smooth; as the tortuosity increases, the fracture surface becomes progressively rougher.

Based on the flow theory of drilling fluid and the deformation theory of fractures, the flow behavior of drilling fluid follows the Navier–Stokes equations (N–S equations). The solution is obtained by assuming that the flow satisfies the continuity equation, the momentum equation, the fluid constitutive equation, and the fracture wall filtration equation.

The continuity equation is expressed as:

(2) $$\frac{\partial \rho }{\partial t}+\nabla \cdot (\rho V)=0$$

where ρ is the fluid density, t is time, V is the volume of the fluid element, and ∇ is the hamilton operator.

The momentum equation is:

(3) $$\rho \frac{Dv}{Dt}=\nabla \cdot P+\rho f$$

where P is the force per unit area (stress tensor), and f is the body force per unit mass.

The fluid constitutive equation (Herschel–Bulkley model) is expressed is:

(4) $$\tau =\tau_{y}+K\gamma^{n}$$

where τ is the shear stress (Pa), $\tau_{y}$ is the yield stress of the model (Pa), K is the consistency coefficient, γ is the shear rate (s^−1^), and n is the flow behavior index.

The overall filtration at the fracture surface is mainly governed by the filtration coefficients controlled by drilling fluid viscosity $C_{v}$, formation fluid viscosity $C_{c}$ and the wall-building ability of the drilling fluid $C_{w}$. The expressions for each filtration coefficient are as follows [35]:

(5) $$C_{v}=0.0469(\frac{K_{m}\Delta p\phi }{\mu_{pv}})$$

where $K_{m}$ is the formation permeability, Δp is the pressure difference between the fluid in the fracture and the formation fluid, ϕ is the formation porosity, and $\mu_{pv}$ is the plastic viscosity of the drilling fluid.

(6) $$C_{c}=0.0374\Delta p\sqrt{\frac{K_{m}c_{f}\Delta p}{\mu_{f}}}$$

where $c_{f}$ is the compressibility of the formation fluid, and $\mu_{f}$ is the viscosity of the formation fluid.

(7) $$C_{w}=0.016\sqrt{\frac{2.25K_{f}c\phi \Delta p}{\mu }}$$

where $K_{f}$ is the permeability of the filter cake, c is the compressibility of the filtrate, ϕ is the porosity of the filter cake, and μ is the plastic viscosity of the filtrate.

The overall filtration coefficient is expressed as:

(8) $$C_{t}=\frac{1}{1/C_{c}+1/C_{v}+1/C_{w}}$$

During the flow of drilling fluid, the pressure within the fracture changes, leading to a reduction in the effective stress on the fracture. This induces deformation, causing the fracture to open or propagate, and results in changes in both fracture width and length.

The fracture deformation index is expressed as [36]:

(9) $$w=w_{0}e^{-\frac{1}{3}\beta (\sigma_{n}-\alpha \cdot p_{f})}$$

where $w_{0}$ is the crack width when the normal stress of the crack is zero, m; α is the Biot coefficient, typically ranging from 0.5 to 1, with smoother cracks having a value closer to 1, dimensionless; $p_{f}$ is the fluid pressure, MPa; $\sigma_{n}$ is the normal stress on the crack surface, MPa; β is an empirical coefficient, obtained through experiments, dimensionless.

A one-dimensional radial loss model is adopted in this study, with the following assumptions: (1) a single rough fracture exists near the wellbore, where drilling fluid filtration occurs at the fracture surface, and the wall filtration is controlled solely by the drilling fluid viscosity; (2) the fluid is an incompressible Herschel–Bulkley (H–B) fluid; (3) the flow of drilling fluid within the fracture is laminar, with no slip at the fracture walls; and (4) the fracture width is much smaller than the fracture propagation length, and the fluid velocity in the radial r direction is much greater than that in the axial z and circumferential θ directions.

**Figure 2:**
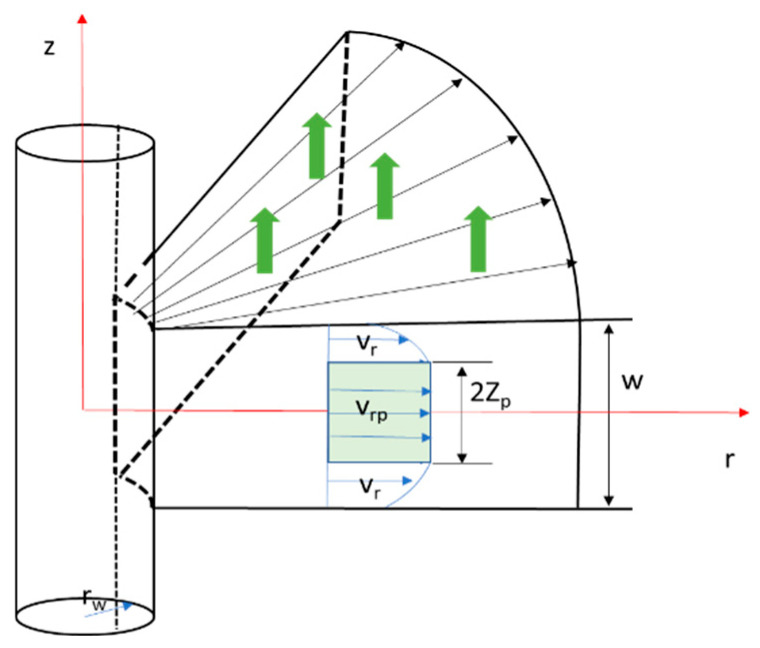
One-Dimensional Radial Lost Circulation Pressure Model.

The one-dimensional radial loss mathematical model is illustrated in Figure 2, where w is the fracture width, $r_{w}$ is the wellbore radius, $v_{rp}$ is the fluid velocity within the flow core, and $v_{r}$ is the fluid velocity outside the flow core.

For one-dimensional radial flow, the mass conservation equation is expressed as:

(10) $$-\frac{1}{r}\frac{\partial }{\partial r}(\rho rwv)=2\rho q_{l}+\frac{\partial \rho w}{\partial t}$$

For an incompressible fluid, the continuity equation is expressed as:

(11) $$-\frac{1}{r}\frac{\partial }{\partial r}(rwv)=2q_{l}+\frac{\partial w}{\partial t}$$

When the drilling fluid flow in the fracture is one-dimensional radial, the velocities in the Z and θ directions and the gravitational force in the Z direction can be neglected. Under this condition, the Navier–Stokes (N–S) equations can be simplified as follows:

(12) $$\rho v\frac{\partial v}{\partial r}=-\frac{\partial p}{\partial r}-[\frac{\partial }{\partial r}(r\tau_{rr})-\frac{\tau_{\theta \theta }}{r}+\frac{\partial \tau_{rz}}{\partial z}]$$

Under low Reynolds number flow, neglecting fluid inertia and assuming that the velocity variation in the radial r direction is smaller than that in the axial z direction, the N–S equations can be further simplified as follows:

(13) $$\frac{\partial p}{\partial r}=-\frac{d\tau_{rz}}{dz}$$

After integration, it can be rewritten as:

(14) $$\tau_{rz}=-\frac{d_{p}}{d_{r}}z$$

In this study, the polymer-based drilling fluid is regarded as a more accurate Herschel–Bulkley (H–B) fluid, and the constitutive equation in the model can be rewritten as:

(15) $$\tau_{rz}=\tau_{z}+K{\left(-\frac{dv_{r}}{dz}\right)}^{n}$$

where $\tau_{rz}$ is the shear stress, Pa; $\tau_{z}$ is the yield stress of the model, Pa; K is the consistency coefficient; $v_{r}$ is the shear rate, s^−1^ and n is the flow behavior index.

By combining Equations (13) and (15), we obtain:

(16) $$-dv_{r}={\left[\frac{1}{K}(-\frac{dp}{dx}y-\tau_{z})\right]}^{\frac{1}{n}}dz$$

(17) $$v_{r}=-\int {\left[\frac{1}{K}(-\frac{dp}{dr}z-\tau_{z})\right]}^{\frac{1}{n}}dz$$

(18) $$v_{r}=-{\left(-\frac{dp}{dr}\right)}^{-1}(\frac{n}{n+1}){\left(\frac{1}{K}\right)}^{\frac{1}{n}}{\left(-\tau_{z}-\frac{dp}{dr}z\right)}^{\frac{n+1}{n}}-C$$

Assuming laminar flow of the drilling fluid and no slip at the fracture walls, we have:

(19) $$v_{r}=0\;,\;z=\pm \frac{w}{2}$$

The constant C and the radial flow velocity $v_{r}$ are given by:

(20) $$C=-{\left(-\frac{dp}{dr}\right)}^{-1}(\frac{n}{n+1}){\left(\frac{1}{K}\right)}^{\frac{1}{n}}{\left(-\tau_{z}-\frac{dp}{dr}\frac{w}{2}\right)}^{\frac{n+1}{n}}$$

(21) $$v_{r}={\left(-\frac{dp}{dr}\right)}^{-1}(\frac{n}{n+1}){\left(\frac{1}{K}\right)}^{\frac{1}{n}}-[{\left(-\tau_{z}-\frac{dp}{dr}z\right)}^{\frac{n+1}{n}}+{\left(-\tau_{z}-\frac{dp}{dr}\frac{W}{2}\right)}^{\frac{n+1}{n}}]$$

Due to the yield stress of the H–B fluid, a plug flow region exists during fluid motion. Assuming that the plug flow radius within the fracture is $Z_{P}$, we have:

(22) $$\tau_{z}=-\frac{dp}{dr}z_{p}$$

(23) $$V_{rp}={\left(-\frac{dp}{dr}\right)}^{-1}(\frac{n}{n+1}){\left(\frac{1}{K}\right)}^{\frac{1}{n}}{\left(-\tau_{z}-\frac{dp}{dr}\frac{W}{2}\right)}^{\frac{n+1}{n}}$$

The total flow rate q of the fracture section drilling fluid is the sum of the flow rate $q_{rp}$ in the flow core and the flow rate $q_{r}$ outside the flow core

(24) $$q_{rp}=2\pi r\int_{-\frac{z_{p}}{2}}^{\frac{z_{p}}{2}}V_{rp}dz=2\pi r\tau_{z}{\left(-\frac{dp}{dr}\right)}^{-2}(\frac{n}{n+1}){\left(\frac{1}{K}\right)}^{\frac{1}{n}}{\left(-\tau_{z}-\frac{dp}{dr}\frac{w}{2}\right)}^{\frac{n+1}{n}}$$

(25) $$\begin{array}{ll} q_{r}=2\pi r\int_{-\frac{w}{2}}^{-\frac{z_{p}}{2}}V_{r} & dz+2\pi r\int_{\frac{z_{p}}{2}}^{\frac{w}{2}}V_{r}dz\\ & =2\pi r{\left(-\frac{dp}{dx}\right)}^{-2}(\frac{n}{2n+1}){\left(\frac{1}{K}\right)}^{\frac{1}{n}}{\left(-\tau_{y}-\frac{dp}{dx}\frac{w}{2}\right)}^{\frac{2n+1}{n}} \end{array}$$

(26) $$\begin{array}{ll} q=q_{rp}+q_{r}= & 4\pi r{\left(\frac{1}{K}\right)}^{\frac{1}{n}}(\frac{n}{2n+1}){\left(\frac{W}{2}\right)}^{\frac{1+2n}{n}}{\left(-\frac{dp}{dr}\right)}^{-2}{\left(-\tau_{z}-\frac{dp}{dr}\frac{W}{2}\right)}^{\frac{n+1}{n}}(\frac{n}{n+1}\tau_{z}\\ & -\frac{dp}{dr}\frac{W}{2}) \end{array}$$

The average velocity of the H–B drilling fluid within the fracture is then given by:

(27) $$\begin{array}{ll} \bar{v}=\frac{q}{A}=\frac{q}{2\pi rw}= & {\left(\frac{1}{K}\right)}^{\frac{1}{n}}(\frac{n}{2n+1}){\left(\frac{W}{2}\right)}^{\frac{1+n}{n}}{\left(-\frac{dp}{dr}\right)}^{-2}{\left(-\tau_{z}-\frac{dp}{dr}\frac{W}{2}\right)}^{\frac{n+1}{n}}(\frac{n}{n+1}\tau_{z}\\ & -\frac{dp}{dr}\frac{W}{2}) \end{array}$$

Assuming the drilling fluid is incompressible, its continuity equation consists of the flow term, fracture deformation term, and wall filtration term. The flow term can be expressed as:

(28) $$\frac{1}{\;r}\frac{\partial }{\partial r}(rwv)=(\frac{n}{2n+1})\frac{1}{2^{1+\frac{1}{n}}\frac{1}{k^{n}}}\{\frac{\partial }{\partial r}[w^{2+\frac{1}{n}}{\left(-\frac{dp}{dr}-\frac{2n+1}{n+1}\frac{2\tau_{z}}{w}\right)}^{\frac{1}{n}}]\;+\frac{1}{r}[w^{2+\frac{1}{n}}{\left(-\frac{dp}{dr}-\frac{2n+1}{n+1}\frac{2\tau_{z}}{w}\right)}^{\frac{1}{n}}]\}\;$$

The fracture deformation index formula can more accurately describe the variation in fracture width with the pressure inside the fracture [37,38,39]. By introducing the fracture tortuosity to characterize fracture roughness in Equation (1). For convenience, setting α = 1, the rough fracture deformation term can be expressed as:

(29) $$w=\delta^{\frac{1}{3}}w_{0}e^{-\frac{1}{3}\beta (\sigma_{n}-p_{f})}$$

Taking the partial derivative with respect to time, we have:

(30) $$\frac{\partial w}{\partial t}=\delta^{\frac{1}{3}}\beta w_{0}e^{-\beta (\sigma -p_{f})}\frac{\partial p_{f}}{\partial t}$$

Assuming that wall filtration is controlled solely by the drilling fluid viscosity, we have:

(31) $$C_{t}=C_{v}=0.0469(\frac{K_{m}\Delta p\phi }{\mu_{pv}})$$

The filtration term can then be expressed as:

(32) $$q_{l}=\frac{2C_{t}}{\sqrt{t-\tau }}=\frac{2C_{v}}{\sqrt{t-\tau }}$$

By substituting Equations (28), (30), and (32) into the continuity equation for incompressible fluid (Equation (11)), the partial differential equation governing the flow of incompressible H–B drilling fluid in a one-dimensional radial fracture is obtained:

(33) $$\begin{array}{c} \left(\frac{n}{2n+1})\frac{1}{2^{1+\frac{1}{n}}k^{\frac{1}{n}}}\{\frac{\partial }{\partial r}[w^{2+\frac{1}{n}}{\left(-\frac{dp}{dr}-\frac{2n+1}{n+1}\frac{2\tau_{z}}{w}\right)}^{\frac{1}{n}}]+\frac{1}{r}[w^{2+\frac{1}{n}}{\left(-\frac{dp}{dr}-\frac{2n+1}{n+1}\frac{2\tau_{z}}{w}\right)}^{\frac{1}{n}}]\right\}\\ +\delta^{\frac{1}{3}}\beta w_{0}e^{-\beta (\sigma -p_{f})}\frac{\partial p_{f}}{\partial t}+\frac{0.1876p_{f}K_{m}\phi }{\mu_{pv}\sqrt{t-\tau }}r\frac{dp}{dr}=0 \end{array}$$

By taking the flow behavior index *n* = 1, Equation (33) can be simplified as:

(34) $$\begin{array}{c} \frac{1}{12k}\{\frac{\partial }{\partial r}[{\left(\delta^{\frac{1}{3}}w_{0}e^{-\frac{1}{3}\beta (\sigma_{n}-p_{f})}\right)}^{3}(-\frac{dp}{dr}-\frac{3\tau_{z}}{w\delta^{\frac{1}{3}}w_{0}e^{-\frac{1}{3}\beta (\sigma_{n}-p_{f})}})]+\\ \frac{1}{r}[{\left(\delta^{\frac{1}{3}}w_{0}e^{-\frac{1}{3}\beta (\sigma_{n}-p_{f})}\right)}^{3}(-\frac{dp}{dr}-\frac{3\tau_{z}}{\delta^{\frac{1}{3}}w_{0}e^{-\frac{1}{3}\beta (\sigma_{n}-p_{f})}})]\}\\ +\delta^{\frac{1}{3}}\beta w_{0}e^{-\beta (\sigma_{n}-p_{f})}\frac{\partial p_{f}}{\partial t}+\frac{0.1876p_{f}K_{m}\phi }{\mu_{pv}\sqrt{t-\tau }}r\frac{dp}{dr}=0 \end{array}$$

### 3.2. Model Validation

#### 3.2.1. Initial and Boundary Conditions of the Model

(1) Initial Conditions

Assuming that the initial pressure within the fracture equals the formation pressure:

(35) $$p_{f}=p_{0}\;,\;\;r=r_{w}\;,\;\;t=0$$

where $p_{0}$ is the formation pore pressure, MPa; $r_{w}$ is the wellbore radius, m.

(2) Boundary Conditions

(36) $$\frac{\partial p}{\partial r}=0\;,\;r=r_{ext}$$

where $r_{ext}$ is the radius at the fracture tip, m.

#### 3.2.2. Model Verification

To verify the rationality of the model, the loss parameters from the target well area were selected for validation. The drilling fluid used onsite had a consistency coefficient of 0.4 Pa·s^n^ and a yield stress of 5 Pa. The fracture tortuosity was taken as 1.4, the average formation porosity as 2%, and the average permeability as 3.54 × 10^−3^ µm^2^.

Based on the loss model, MATLAB simulations were conducted to calculate the loss rates under the actual pressure differentials at each loss point. The results are shown in Table 3. The total error is 8.5%, which meets engineering requirements, indicating that the established fracture loss pressure prediction model is feasible for the target well area.

#### 3.2.3. Initial Parameters of the Maokou Formation Shale Model

Based on field engineering data, laboratory rock mechanics experiments, well logging interpretation results, and literature sources [40,41,42,43,44], the initial parameters of the H–B fluid drilling fluid fracture loss pressure model were determined, as shown in Table 4.

### 3.3. Analysis of Drilling Fluid Loss Patterns in Fractured Formations

Based on the lost circulation pressure calculation model, this study comprehensively analyzed the effects of fracture characteristic parameters, drilling fluid properties, fracture stress state, and wellbore–formation pressure differential on fracture-induced loss in the Maokou Formation shale using the control variable method. Additionally, employing a loss classification approach, the types of loss under different parameter conditions were categorized.

#### 3.3.1. Influence of Fracture Characteristic Parameters on Lost Circulation

**Figure 3:**
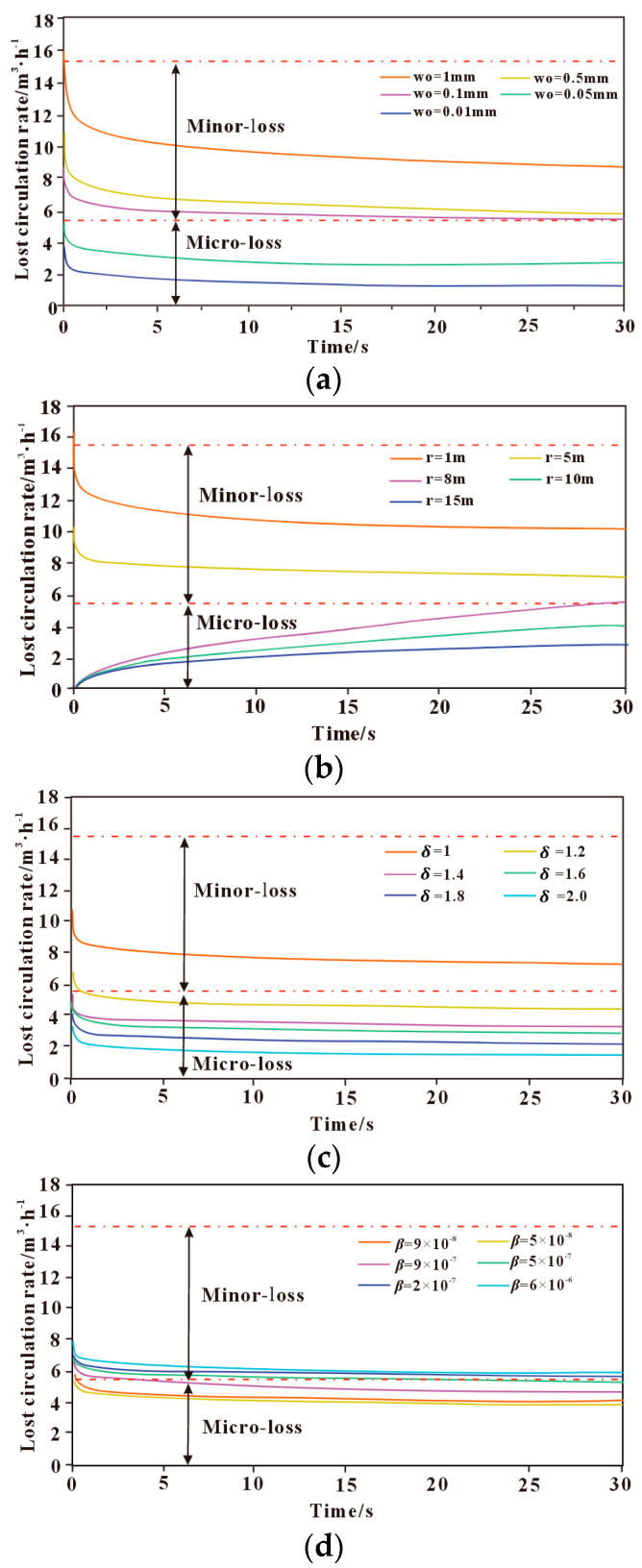
The influence of different fracture characteristic parameters on the leakage law. (**a**) Initial crack width; (**b**) Radial length of crack; (**c**) Fracture roughness; (**d**) Crack index deformation coefficient.

The influence of fracture characteristic parameters on drilling fluid loss mainly includes fracture width, fracture length, fracture roughness, and fracture deformation index. The effects of these fracture characteristic parameters on the lost circulation behavior are shown in Figure 3. As shown in Figure 3a, the drilling fluid loss rate reaches its maximum at the early stage of loss, then gradually decreases and tends to stabilize. Moreover, the loss rate increases with the enlargement of the initial fracture aperture; a wider fracture provides a larger flow channel for drilling fluid, thereby increasing the lost circulation rate. When the initial fracture width changes, the drilling fluid loss rate varies significantly, indicating that the initial fracture width has a strong influence on lost circulation. When the initial fracture width is greater than 1 mm, the initial leakage rate of drilling fluid is greater than 15 m^3^/h, which belongs to medium leakage. When the initial crack width is between 0.1 mm~1 mm, the leakage type is small leakage; when the initial crack width is less than 0.1 mm, the leakage type is micro leakage. When the fracture width is small, the yield behavior of the polymer drilling fluid is more pronounced, resulting in greater flow resistance within the fracture and a lower loss rate. As the fracture width gradually increases, the shear rate of the fluid rises and the viscosity decreases, leading to a gradual increase in the loss rate.

As shown in Figure 3b, under the same initial fracture width, the drilling fluid loss rate decreases with increasing radial extension length of the fracture. When the fracture extension length is less than 5 m, the drilling fluid loss rate reaches a peak at the onset of loss, then gradually decreases and approaches a steady state, with the loss type classified as slight-to-moderate loss. When the fracture extension length exceeds 5 m, a larger extension length results in a smaller pressure gradient within the fracture. The initial loss rate tends to approach zero, then gradually increases over time, but the overall loss rate remains relatively low, which is classified as micro-loss to slight loss. The fracture radial extension length not only affects the fracture conductivity but also alters the distribution characteristics of the fracture pressure gradient. As the radial extension length increases, the cumulative frictional resistance acting on the fluid becomes more significant, resulting in a gradual decrease in the pressure gradient along the fracture. Consequently, the shear rate of the polymer drilling fluid decreases, and the loss rate gradually diminishes.

As shown in Figure 3c, the lost circulation rate decreases with increasing fracture tortuosity, indicating that the rougher the fracture surface, the lower the loss rate. The loss rate reaches its peak at the onset of fluid loss and then gradually decreases until it approaches a steady state. When the fracture tortuosity is greater than 1.4, the loss type is classified as micro-loss, whereas when the tortuosity is less than 1.4, the loss type is classified as slight loss. With the increase in fracture tortuosity, the fracture surface roughness gradually increases, leading to a longer and more complex flow path of the fluid within the fracture. This results in greater local energy loss and a gradual reduction in the loss rate.

As shown in Figure 3d, the larger the fracture deformation index, the more easily the fracture deforms. The lost circulation rate increases with an increasing fracture deformation index, reaching its peak at the onset of fluid loss and then gradually decreasing until it stabilizes. Under different fracture deformation indices, the loss type falls between micro-loss and slight loss.

Overall, it can be seen that different fracture characteristic parameters have varying degrees of influence on the drilling fluid loss rate. The geometric characteristics of the fracture influence the yield behavior of the polymer fluid by altering the fracture conductivity and the internal flow conditions within the fracture, thereby forming a “fracture geometry–fluid rheology” coupling control mechanism. The initial fracture width and radial extension length have a relatively strong influence on the drilling fluid loss rate, whereas the effect of fracture roughness on the loss rate is comparatively smaller than that of the initial fracture width and radial extension length. The fracture deformation index has the least influence on the drilling fluid loss rate, indicating that it is not a primary controlling factor of fracture-induced lost circulation. Therefore, it is of great significance to adopt corresponding drilling fluid optimization measures and lost circulation control strategies tailored to different fracture characteristics.

#### 3.3.2. Influence of Drilling Fluid Properties on Lost Circulation

**Figure 4:**
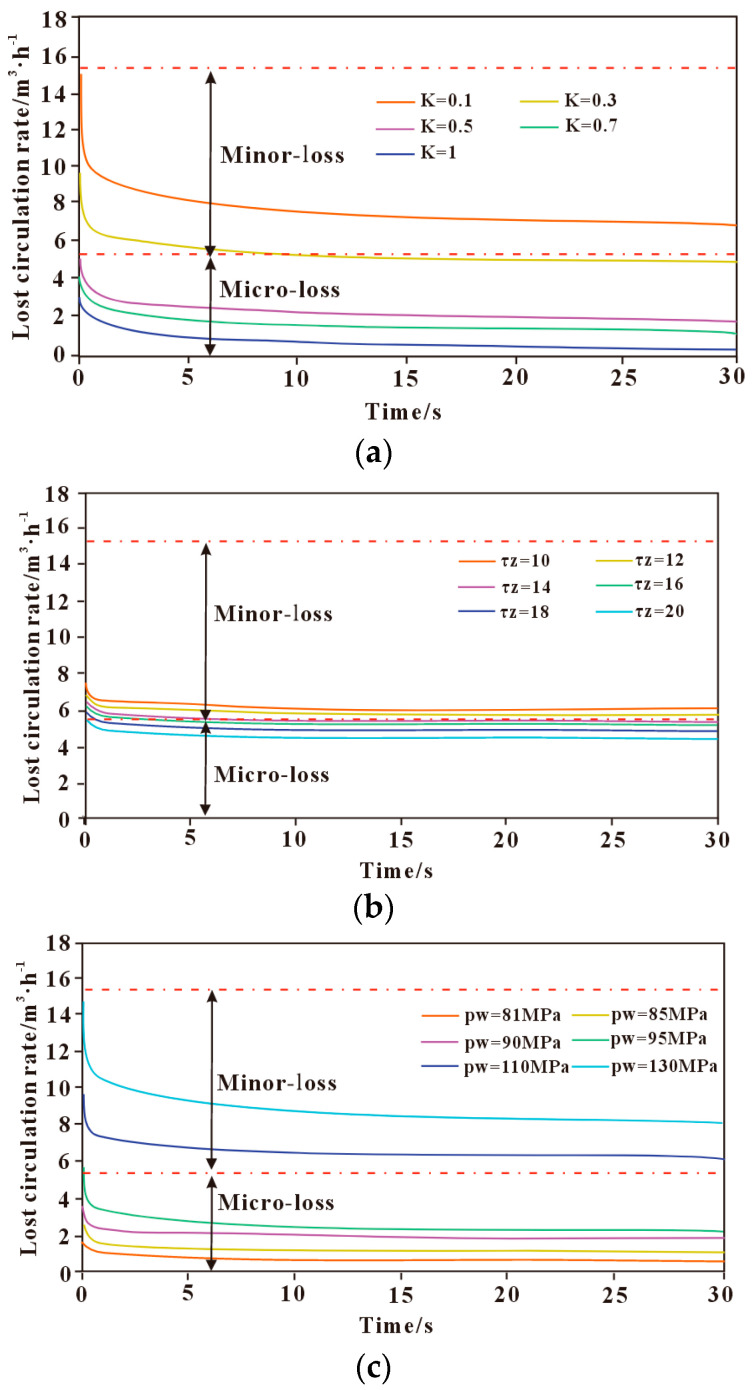
The influence of different drilling fluid properties on the leakage law. (**a**) Consistency coefficient; (**b**) Dynamic shear force; (**c**) Wellbore pressure.

The influence of drilling fluid properties on lost circulation behavior mainly involves the consistency coefficient, dynamic yield stress, and drilling fluid density (wellbore pressure pw). The effects of different polymer-based drilling fluid properties on lost circulation behavior are shown in Figure 4. As shown in Figure 4a, the lost circulation rate decreases with an increasing consistency coefficient. The loss rate is highest at the onset of fluid loss and gradually decreases until reaching a steady state. The smaller the consistency coefficient, the faster the change in loss rate over time. When the consistency coefficient is less than 0.3 Pa·s^n^, the loss type is classified as slight loss, whereas when it is greater than 0.3 Pa·s^n^, the loss type is classified as micro-loss.

As shown in Figure 4b, the lost circulation rate decreases with increasing dynamic yield stress. The loss rate is highest at the onset of fluid loss and gradually decreases until it reaches a steady state. With changes in dynamic yield stress, the variation in loss rate is relatively small, indicating that dynamic yield stress has a limited effect on fracture-induced lost circulation. When the dynamic yield stress is less than 12 Pa, the loss type is classified as slight loss but close to micro-loss; when it exceeds 12 Pa, the loss type is classified as micro-loss.

As shown in Figure 4c, the greater the wellbore pressure, the higher the lost circulation rate, and the faster the rate changes over time. The loss rate peaks at the onset of lost circulation, then gradually decreases and approaches a steady state. When the pressure differential is in the range of 1–15 MPa, the loss rate is relatively low and classified as micro-loss, whereas when the pressure differential exceeds 15 MPa, the loss type is classified as slight loss.

The greater the consistency coefficient and yield stress of the polymer-based drilling fluid, the stronger its rheological characteristics, resulting in increased flow resistance within the fracture and a gradual reduction in the loss rate. When the wellbore pressure increases, the pressure differential driving force within the fracture also increases, leading to a gradual rise in the fracture loss rate. In addition, the rheological properties of the polymer-based drilling fluid not only determine its flow resistance within the fracture but also interact with fracture roughness and deformation, affecting the spatial distribution of local shear rate and filtrate loss rate. Different drilling fluid property parameters have a significant effect on the lost circulation rate, with the consistency coefficient and drilling fluid density exerting a greater influence, while the impact of dynamic yield stress is relatively minor. During drilling operations, selecting an appropriate drilling fluid is particularly important to prevent drilling fluid loss and minimize potential damage to the formation.

#### 3.3.3. Influence of Fracture Stress State on Lost Circulation

**Figure 5:**
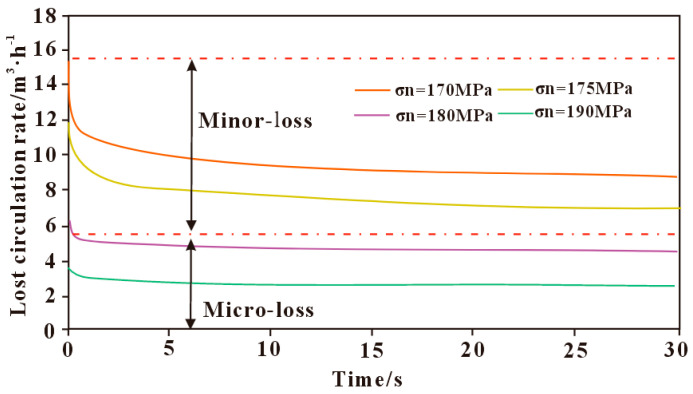
Effect of fracture stress state on leakage.

The model in this study considers horizontal fractures in a vertical well, where the fracture normal stress is controlled by the overburden pressure. The variation in drilling fluid loss patterns under different normal stresses is shown in Figure 5. It can be observed that the loss rate decreases with increasing normal stress, and the higher the normal stress, the smaller the change in loss rate. This is mainly because a higher normal stress results in a smaller fracture aperture, leading to greater frictional resistance for the polymer drilling fluid during flow within the fracture. The peak loss rate occurs at the onset of loss, then gradually decreases and stabilizes. The loss rate varies significantly under different normal stresses, indicating that normal stress has a considerable influence on fracture-induced loss. Specifically, when the normal stress is less than 170 MPa, the loss is classified as moderate; when it is between 170 MPa and 180 MPa, the loss is minor; and when it exceeds 180 MPa, the loss is micro-loss.

#### 3.3.4. Influence of Wellbore–Formation Pressure Differential on Loss Rate

**Figure 6:**
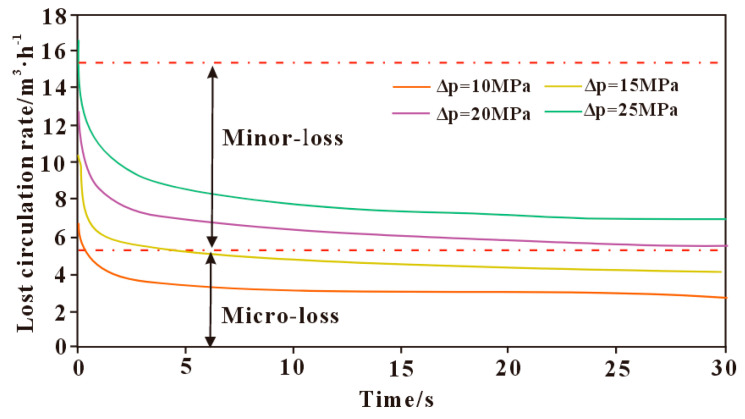
Effect of pressure difference between wellbore and formation on leakage rate.

The influence of the wellbore–formation pressure differential on the loss rate is shown in Figure 6. It can be seen that an increase in bottom-hole pressure differential results in a significant change in the drilling fluid loss rate. At the initial stage of loss, the loss rate reaches a peak, then gradually stabilizes as the loss progresses. The pressure differential between the wellbore and formation is one of the main driving forces for drilling fluid loss; the greater the differential, the more severe the loss. Excessive pressure differential can cause fracture opening and extension, providing a larger pathway for fluid loss and exacerbating the loss. When the pressure exceeds the fracture pressure, it may even lead to fracturing-induced loss.

**Figure 7:**
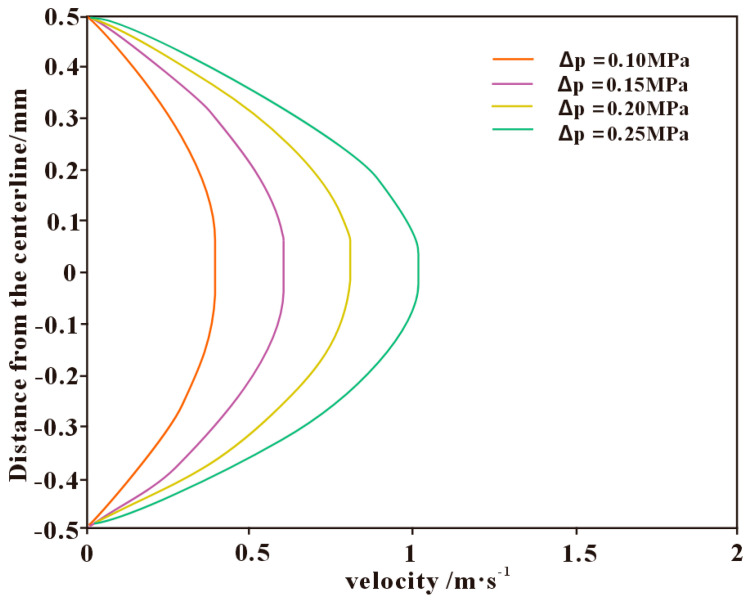
Velocity distribution of drilling fluid within the fracture under different fracture pressure drops.

When the pressure difference between the wellbore and the formation is in the range of 10–25 MPa, the calculated actual pressure drop within the fracture is approximately 0.1–1 MPa. The velocity distribution of the drilling fluid within the fracture under different fracture pressure drops is shown in Figure 7. The fluid inside the fracture exhibits a typical two-layer structure: the inner region is characterized by plug flow, while the outer region corresponds to a parabolic-shaped shear flow. As the pressure drop increases, the overall flow velocity in the fracture gradually rises, and the plug-flow zone becomes progressively thinner. When the pressure drop within the fracture reaches approximately 0.25 MPa, the plug-flow region nearly disappears, and the flow regime transitions from a combined “plug–shear” pattern to a single “shear-flow” pattern. During the flow of polymer-based drilling fluids within fractures, a larger plug-flow thickness helps reduce the infiltration rate of the fluid, thereby mitigating the loss behavior. Therefore, in deep fractured shale formations, reasonably controlling the pressure differential to maintain an appropriate proportion of plug flow is of great significance for achieving effective wellbore sealing and loss prevention.

### 3.4. Application of Polymer-Based Drilling Fluids

Rheological properties are one of the key characteristics of drilling fluids, playing an essential role in cuttings transport, reducing circulating pressure losses, and enhancing the rate of penetration. At present, many drilling fluids are formulated with polymers as primary additives or employ polymers to regulate their main properties; such fluids are referred to as polymer-based drilling fluids. Polymers mainly influence lost circulation behavior by adjusting the fluid viscosity and improving its consistency index and flow behavior. Commonly used polymers include natural polymers such as cellulose-based, starch-based, and polysaccharide-based polymers, as well as synthetic polymers such as polyacrylamides and hydrolyzed polyacrylonitriles. With increasing drilling depth, formation temperatures rise continuously, placing higher demands on the performance of drilling fluids. Under high-temperature conditions, natural polymers such as cellulose- and starch-based polymers are prone to hydrolysis or oxidation, leading to reduced stability of the drilling fluid. In contrast, synthetic polymers, such as polyacrylamides, are more resistant to degradation at elevated temperatures, with relatively stable molecular chains [45,46].

**Figure 8:**
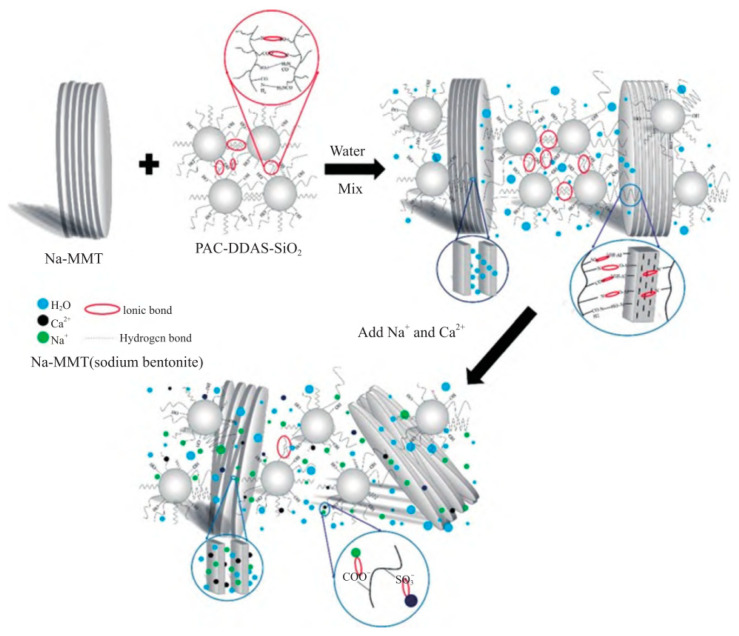
Fluid loss reduction interaction mechanism of PAC-DDAS-SiO_2_ [46]. MMT: montmorillonite; PAC-DDAS: a kind of copolymer.

To address the issue of insufficient stability of natural polymers under high-temperature conditions, natural macromolecules are typically processed to the nanoscale and subsequently modified to enhance their temperature and salt resistance. Numerous studies have demonstrated that the incorporation of nanomaterials plays a significant role in improving the thermal stability of polymers [47,48]. Figure 8 illustrates the fluid-loss reduction mechanism of the polymer-based drilling fluid. It can be observed that the spherical copolymer particles possess side chains containing carboxyl, sulfonic, and amide groups. Two side chains within the same particle, as well as those from different particles, can interact through ionic and hydrogen bonding. These interactions cause the copolymer particles to arrange compactly and stack with each other, forming a self-assembled structure with interconnected linear and planar networks. Through adsorption and encapsulation of clay particles, the copolymer enhances the dispersion of the clay. Moreover, the nanoscale effect and self-assembled structure of the copolymer increase the thickness of the hydration film on the clay particle surface, thereby improving the quality of the filter cake and reducing the fluid loss.

In addition, interactions between different polymers may produce synergistic effects. For example, a calcium-polymer drilling fluid, in which a sulfonated polymer acts as a filtrate reducer, can enhance the thermal stability of starch when used together, increasing its stability up to 145 °C [49]. Different polymers can significantly influence the properties of drilling fluids. During drilling, appropriate polymers can be selected based on the geological conditions of the formation to formulate drilling fluids, thereby achieving more efficient and safer drilling operations.

## 4. Conclusions

This study addresses the problem of drilling fluid loss during deep fracture-prone shale drilling, taking the Maokou Formation shale in the southern Sichuan Basin as an example. The variations in rock mechanical properties under different soaking durations were systematically analyzed. Based on the traditional model, a drilling fluid loss pressure calculation model was established by introducing the yield stress term of the polymer fluid and considering the effects of fracture roughness, conductivity variation, and the filtrate seepage process, taking into account the fracture geometric characteristics, drilling fluid properties, fracture stress state, and the wellbore–formation pressure differential. The effects of variations in these parameters on the drilling fluid loss rate were investigated, and the results indicate the following:

(1) Contact between drilling fluid and shale reduces the uniaxial compressive strength and elastic modulus of the rock, potentially leading to wellbore instability and related incidents. Although the Maokou Formation shale is dominated by illite, which has weak hydration and swelling potential, drilling fluid still significantly weakens the mechanical strength of the rock.
(2) The drilling fluid loss rate increases with increasing initial fracture width and fracture deformation index, and decreases with increasing fracture radial extension length and fracture roughness. Among these factors, the fracture deformation index has a relatively small impact on the loss pattern and is not a controlling factor for fracture-induced loss.
(3) The drilling fluid loss rate increases with increasing consistency coefficient and yield stress, and decreases with increasing drilling fluid density. Among these factors, the yield stress has a relatively small influence on the loss rate.
(4) The drilling fluid loss rate decreases with increasing fracture normal stress and increases with increasing wellbore–formation pressure differential.

In summary, drilling fluid loss is the result of multiple interacting factors, including fracture characteristics, formation stress state, drilling fluid properties, and the wellbore–formation pressure differential. To effectively control fluid loss, the influence of each factor must be considered, and appropriate measures should be implemented to reduce the loss rate. The findings of this study provide a theoretical basis and technical support for lost circulation control in deep fracture-prone shale formations.

## Figures and Tables

**Table 1:** XRD Analysis of Clay Minerals in Maokou Formation Shale.

Mineral Component	Relative Clay Mineral Contents			Ratio of Mixed-Layer
	I/S	It	C	I/S
Mean value	30.9%	47.3%	21.8%	6.1%
Range	29–36%	40–68%	19–23%	5–8%

**Table 2:** Porosity and Permeability of Maokou Formation Shale.

Numbering	D (mm)	H (mm)	Porosity (%)	Permeability (×10^−3^ μm^2^)
C_1_	25.12	25.08	1.38	0.01778
C_2_	25.55	24.93	1.27	0.01693
C_3_	24.62	25.02	1.34	0.01916

**Table 3:** Model Error Analysis.

Loss Pressure Differential (MPa)	Lost Circulation Rate m^3^/h		Error%	
	Actual	Simulation	Single-Point Error	Total Error
28.43	15.8	16.45	5.1	8.5%
26.84	12.6	13.48	11.3	
29.02	17.04	16.45	6	
29.37	12.8	14.9	17.2	
29.45	18.3	19.91	9.3	
30.23	18.8	19.89	5.3	
30.54	21.5	19.83	8.1	
27.35	15.8	14.32	5.6	

**Table 4:** Basic Parameters.

Parameter		Taking Values	Parameter		Taking Values
Formation pressure	p_0_/MPa	80	Porosity	ϕ/%	1
Wellbore pressure	pw/MPa	95	Crack index deformation coefficient	β	9 × 10^−8^
Normal stress	$\sigma_{n}/MPa$	170	Crack tortuosity	δ	1.5
Plastic viscosity	$\mu_{pv}$/Pa·s	40	Initial crack width	$w_{0}$/m	0.5 × 10^−4^
Dynamic shear force	$\tau_{z}$/Pa	10	Radial length of crack	r/m	10
Consistency coefficient	k/Pa·s^n^	0.1	Time step	Δt/s	0.01
Permeability	K_m_/10^−3^ μm^2^	0.5	Simulation time	t/s	30
flow behavior index	*n*	1			

## Data Availability

The original contributions presented in this study are included in the article. Further inquiries can be directed to the corresponding author.
